# Intradialytic Tolerance and Recovery Time in Different High-Efficiency Hemodialysis Modalities

**DOI:** 10.3390/jcm13020326

**Published:** 2024-01-06

**Authors:** Agnieszka Zakrzewska, Jan Biedunkiewicz, Michał Komorniczak, Magdalena Jankowska, Katarzyna Jasiulewicz, Natalia Płonka, Bogdan Biedunkiewicz, Sylwia Małgorzewicz, Agnieszka Tarasewicz, Ewelina Puchalska-Reglińska, Janusz Siebert, Alicja Dębska-Ślizień, Leszek Tylicki

**Affiliations:** 1 Department of Nephrology, Transplantology and Internal Diseases, Medical University of Gdańsk, Smoluchowskiego 17, 80-214 Gdańsk, Polandmichal.komorniczak@gumed.edu.pl (M.K.); magdalena.jankowska@gumed.edu.pl (M.J.); kateolivia@gumed.edu.pl (K.J.); bogdan.biedunkiewicz@gumed.edu.pl (B.B.);; 2 Department of Anesthesiology and Intensive Therapy, Faculty of Medicine, Medical University of Gdańsk, 80-214 Gdańsk, Poland; jan.biedunkiewicz@gumed.edu.pl; 3 Department of Clinical Nutrition, Medical University of Gdańsk, Dębinki 7, 80-211 Gdańsk, Poland; 4 Dialysis Unit, 7th Naval Hospital in Gdańsk, 80-305 Gdańsk, Poland; 5 Department of Family Medicine, University Center for Cardiology, Medical University of Gdansk, 80-211 Gdansk, Poland

**Keywords:** hemodialysis, hemodiafiltration, expanded hemodialysis, quality of life

## Abstract

There are several forms of maintenance high-efficiency hemodialysis (HD), including hemodiafiltrations (HDF) in different technical modes and expanded HD, using dialyzers with medium cut-off membranes. The aim of the study was to assess the intradialytic tolerance and length of dialysis recovery time (DRT) in these modalities. This is an exploratory, crossover study in maintenance HD patients with low comorbidity and no clinical indications for the use of high-efficiency HD, who were exposed to five intermittent dialyses in random order: high-flux hemodialysis (S-HD), expanded HD (HDx), pre-dilution HDF (PRE-HDF), mix-dilution HDF (MIX-HDF) and post-dilution HDF (POST-HDF). Twenty-four dialysis sessions of each method were included in the analysis. Dialysis parameters, including blood flow rate, dialysis fluid flow rate and temperature, and pharmacological treatment were constant. Average total convection volume for post-HDF, pre-HDF and mix-HDF were 25.6 (3.8), 61.5 (7.2) and 47.1 (11.4) L, respectively. During all therapies, patients were monitored for the similarity of their hydration statuses using bioimpedance spectroscopy, and for similar variability over time in systemic blood pressure and cardiac output, while peripheral resistance was monitored using impedance cardiography. The lowest frequency of all intradialytic adverse events were observed during HDx. Delayed DRT was the shortest during PRE-HDF. Patients were also more likely to report immediate recovery while receiving PRE-HDF. These differences did not reach statistical significance; however, the study results suggest that intradialytic tolerance and DRT may depend on the dialysis method used. This supports the need of taking into account patient preferences and quality of life while individualizing high-efficiency therapy in HD patients.

## 1. Introduction

For some time now, hemodialysis (HD) using high-flux membranes is the standard of chronic dialysis treatment (S-HD) replacing dialysis based on low-flux membranes. Technological advances over the past few decades have contributed to further developments in HD therapy and the introduction of high-efficiency dialysis therapies into clinical practice. Significant technological changes in dialyzer membrane permeability and ultrafiltration-controlled delivery systems permitted the more efficient removal of larger–medium-sized water-soluble toxins. There are several forms of high-efficiency dialysis treatment, which include, among others: hemodiafiltration (HDF) in pre-dilution (PRE-HDF), post-dilution (POST-HDF) and mixed dilution (MIX-HDF) mode and the so-called expanded HD (HDx) using dialyzers with medium cut-of membranes (MCO) [1,2,3]. The observational studies and some secondary analyses of randomized trials have indicated that high-volume online HDF may improve patient survival in comparison to S-HD, regardless of whether pre-dilution or post-dilution mode is used [4,5]. Quite recently, the CONVINCE (Comparison of high-dose HDF with high-flux HD) trial confirmed that the use of high-volume POST-HDF resulted in a lower risk of death from any cause than conventional S-HD [6]. Pending the results of other controlled studies in this area, this method is being used increasingly, especially in patients with high comorbidity, long duration of dialysis therapy and contraindications to kidney transplantation [7]. Some experts recommended the use of high-volume online POST-HDF in patients whose Age-Adjusted Charlson Comorbidity Index (AACCI) is ≥8 [8]. Particularly, clinical benefits have been demonstrated in patients with hemodynamic instability, poorly controlled blood pressure (BP), polyneuropathy, calcium–phosphate disorders, pruritus or erythropoietin resistance, among others [3,9]. There is little clinical experience in the use of high-efficiency HD methods in patients with low comorbidity for a chance for a kidney transplant and a potentially short period of dialysis—the vast majority of whom are still dialyzed by classic high-flux HD.

## 2. Materials and Methods

### 2.1. Study Design

This is an exploratory, open, crossover (one-center) study in maintenance HD patients who were exposed to (i) high-flux S-HD and four high-efficiency intermittent dialysis modalities in random order: (ii) HDx, (iii) PRE-HDF, (iv) MIX-HDF, (v) POST-HDF. Each patient underwent three sessions in each of these modalities during one week. The second and third sessions of the week entered the final analysis. Patients and dialysis unit staff were not blinded to treatment allocation. The aim of the study was to compare patients’ tolerance of dialysis methods in a group of patients with low comorbidity who have no clinical indications for the use of high-efficiency dialysis. The study was conducted according to the guidelines of the Declaration of Helsinki, and approved by the Ethical Committee at the Medical University of Gdansk (no. NKBBN/479-759/2022; 18 November 2022).

### 2.2. Patients

The inclusion criteria were as follows: adult patients, eligible for kidney transplantation, treated chronically with HD 3 × per week for at least 6 months; dialysis single-pool Kt/V for urea (spKt/Vurea) > 1.2; patient’s weight in the range of 60–85 kg; AACCI < 8; achievement of a blood flow of >350 mL/min through a fistula or arteriovenous catheter. Exclusion criteria include life expectancy <6 months, severe incompliance to the HD procedures and accompanying prescriptions, emergency hospitalization within 30 days before entering the study, diabetes, active inflammation, active cancer, hemodynamic instability during HD sessions, poorly controlled BP, uremic polyneuropathy, uremic pruritus, dialysis amyloidosis and erythropoietin resistance. Also, patients needed to have no contraindication for bioimpedance measurement and be able to record dialysis recovery time (DRT).

### 2.3. Dialysis Prescription and Equipment

All dialysis therapies were performed on Fresenius 5008 dialysis machine with AutoSub Plus system (Fresenius Medical Care, Bad Homburg, Germany). SHD and HDF treatments were performed with high-flux FX 100 dialyzers (effective surface area: 2.2; UF coefficient 73 mL/h × mmHg; Fresenius Medical Care; Bad Homburg, Germany). HDx sessions were performed using Terranova 400 MCO dialyzer (effective surface area: 1.7 m^2^, UF coefficient 48 mL/h × mmHg; Baxter, Alliston, ON, Canada). Dialysis session time was set at 4 h for all modalities. Temperature of dialysate was set at 36.5 C degree. Blood flow rate and dialysate flow rate were set to 350 and 500 mL/min, respectively. The dry weight of the patients was confirmed before the start of the study using bioimpedance spectroscopy. The fluid removal of each session (ultrafiltration) was set according to individual patient’s interdialytic weight gain plus fluid intake during the procedure and bloodline priming volume. Ultrafiltration profiling and sodium profiling were not used. The electrolyte composition of dialysis fluid was: Na 138–140 mmol/L; K 2.0–3.0 mol/L; HCO_3_ 32 mmol/L; Ca 1.25–1.5 mmol/L; Mg 0.5 mmol/L; Cl 110 mmol/L; glucose 1.0 g/L (10 patients—83.3%: K—2.0 mmol/L; 11 patients—91.7%: Ca—1.25 mmol/L). All patients received standard heparin as a bolus and continuous infusion in accordance with current practice. Sterile and nonpyrogenic substitution fluid for HDF was produced online by ultrafiltration of the ultrapure dialysate. Substitution fluid rate and convection rate during HDF modalities were optimized automatically using the AutoSub Plus system based on pressure pulse attenuation and cross-membrane pressure assessment (Fresenius Medical Care; St. Wendel, Germany). The basic principle of AutoSub Plus is to avoid excessive hemoconcentration in the dialyzer and maximization of the ultrafiltration flow [10]. For a given patient, dialysis settings were kept unchanged during all treatment modalities, e.g., post-dialysis weight, dialysis session length, composition of the dialysis fluid, blood and dialysis fluid flow, dialysis fluid temperature and anticoagulation dose. The patient’s concomitant medications were continued in an unchanged manner.

### 2.4. Outcomes

During all sessions, adverse events (AEs), DRT, hemodynamic parameters and hydration state were recorded. The results from the middle and the last dialysis sessions in weeks were used in the analysis.

#### 2.4.1. Adverse Events

The frequency of symptomatic hypotension, AEs potentially related to BP/fluid shifts, AEs not classically related to BP/fluids shifts and intradialytic clotting events were recorded. Symptomatic hypotension was defined as a decrease in systolic BP ≥ 20 mm Hg, requiring reduction in or cessation of ultrafiltration and/or need for intravenous fluid bolus or head-down tilt of dialysis chair. AEs potentially related to BP/fluid shifts were defined as experiencing breathlessness, cramps (normal BP), dizzy/lightheaded, falling, headache, erratic venous pressures, clotted needle or restless legs. AEs not classically related to BP/fluids shifts were defined as aches in bones, arm pain, back pain, bleeding, constipation, diarrhea, feeling cold, feeling down, feeling hot, generally unwell, heavy legs, increased lethargy, infection (given antibiotics), itch, leg pain, nausea, stomach pains, sweating, swollen abdomen and vomiting. Intradialytic clotting events were defined as either an increase in venous pressures requiring additional anticoagulant dosing or clotting of the extracorporeal circuit [11].

#### 2.4.2. Dialysis Recovery Time

At each dialysis session, the patient was asked the duration of DRT to baseline function, following their antecedent dialysis session. The patients’ responses were converted to a number of minutes, as follows [12]:

i. Answers given in minutes were recorded directly.
ii. Answers in hours were multiplied by 60.
iii. Variants of “half a day”, including the “next day”, were given a value of 720 min.
iv. Variants of “one day” were given a value of 1440 min.
v. Variants of “more than a day” were given a value of 2160 min (36 h).

Given that the distribution of DRT was bimodal with a peak at zero, it was analyzed via separate crossover analysis: percentage of immediate DRT (equal 0 min) and delayed DRT in minutes.

#### 2.4.3. Hemodynamic Monitoring

For real-time hemodynamic measurements, the CardioScreen 2000 (Medis. Medizinische Messtechnik GmbH, Ilmenau, Germany) device was used. CardioScreen 2000 is a feasible and accurate method for non-invasive hemodynamic measurements using methods of impedance cardiography, which utilizes a physiological adaptive signal analysis (PASA) algorithm. Hemodynamic measurements obtained using a PASA algorithm were correlated highly significantly to measurements obtained via the thermodilution method [13]. The following parameters were measured or calculated: systolic BP (SBP), diastolic BP (DBP), mean arterial pressure (MAP), cardiac index (CI), systemic vascular resistance index (SVRI). Hemodynamic parameters were measured in resting position 10 min prior to dialysis, during dialysis (at the following time points: 15, 30, 60, 120, 180, 240 min) and 10 min after dialysis. In order to aggregate the changes in time during the entire dialysis session, the area under the curve (AUC) of BP, CI and SVRI were calculated using the trapezoid method.

#### 2.4.4. Hydration State

Body composition and hydration state had been assessed using a portable whole body bioimpedance spectroscopy device (BCM; Fresenius Medical Care, Bad Homburg, Germany). The measurements were obtained before and after dialysis session in resting position. The extracellular water (ECW), intracellular water (ICW) and total body water (TBW) were calculated from a fluid model [14].

### 2.5. Statistics

Continuous data are reported as means (±standard deviation, SD) or medians (inter-quartile ranges, IQR). The Shapiro–Wilk test was used to determine the distribution of continuous variables. Categorical data are reported as percentages of the total. The Wilcoxon signed-rank test or ANOVA was used in the analysis comparing the results of the variables repeatable more than twice. Two-sided *p* < 0.05 was considered to be statistically significant. The statistical analysis was performed using the program Statistica 13.3 (TIBCO Software Inc.; Palo Alto, CA, USA). Given that the distribution of DRT was bimodal with a peak at zero, it was analyzed via separate analysis with 2 models (immediate DRT as categorical variable and delayed DRT as continuous variable).

## 3. Results

### 3.1. Characteristics of Patients

Twelve patients met the inclusion criteria and were enrolled to the study, eleven of whom were men (91.67%) and one woman (8.33%), with a mean age of 52.5 ± 15.47 years. Hypertension was diagnosed in 10 (83.3%) patients. A description of the study group is presented in Table 1.

### 3.2. Dialysis Parameters

Dialysis session time, blood flow rate and dialysate flow rate were constant during all modalities. All patients achieved the minimum level of convection for high-volume post-HDF with a substitution volume >21 L. Mean (standard deviation) total convection for post-HDF, pre-HDF and mix-HDF were 25.6 (3.8), 61.5 (7.2) and 47.1 (11.4) L, respectively. The target body weight was achieved during all studied dialysis sessions. The fluid removal, SBP, DBP, TBW, ECW and ICW did not differ between tested treatments. Detailed dialysis parameters and patients’ hydration status results are presented in Table 2.

### 3.3. Hemodynamic Parameters

**Figure 1:**
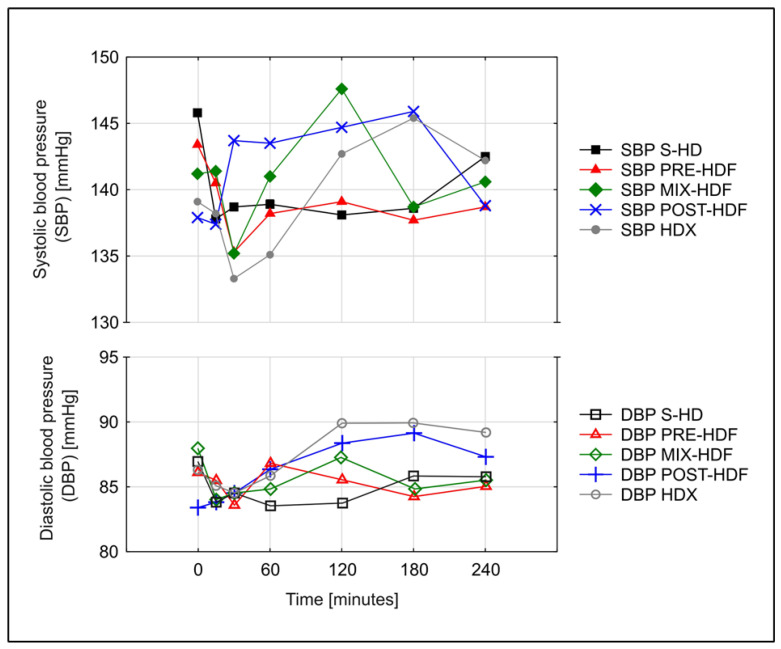
Changes over time in mean systolic (SBP) and diastolic blood (DBP) pressure during various dialysis treatments.

**Figure 2:**
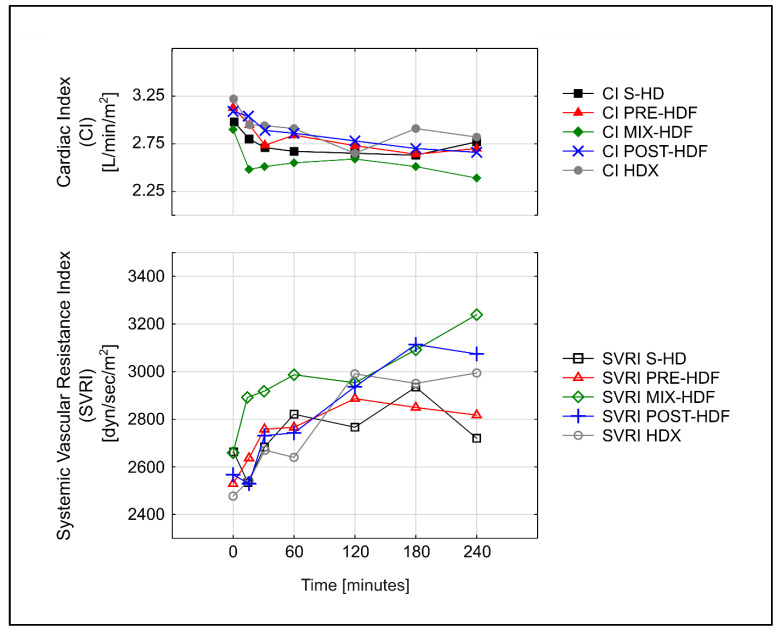
Mean area under the curve (AUC) of changes over time in cardiac index (CI) and systemic vascular resistance index (SVRI) during various treatments.

SBP and DBP at the beginning (first minute) and at the end of dialysis (240 min) sessions did not differ between treatments. AUC of SBP, DBP and MAP measurements obtained during dialysis over time did not differ between treatments as well. CI was decreasing (*p* < 0.001 for all methods) while SVRI was increasing (*p* < 0.001 for all methods) during all methods used. The AUC of CI and SVRI measurements obtained during dialysis over time did not differ between the treatments. Detailed results are presented in Table 3 and Figure 1 and Figure 2.

### 3.4. Adverse Events and Dialysis Recovery Time

AEs were grouped to those that may or may not have been related to BP changes or fluid shifts and those related to clotting events. There were no incidents of symptomatic intradialytic hypotension during any treatment. The lowest frequency of all AEs was observed with HDx (25%), although the differences did not prove to be statistically significant. Delayed DRT was the shortest during PRE-HDF. Patients were also more likely to report immediate recovery while receiving PRE-HDF (62.5%). However, the differences did not reach statistical significance. Detailed results are presented in Table 4.

## 4. Discussion

The CONVINCE trail provides the first convincing evidence that patients receiving high-volume POST-HDF have improved survival compared with those receiving high-flux HD [6]. It appears to be a milestone that indicates the therapy of choice for patients treated with long-term dialysis [15]. However, the question of what therapy to offer patients with low comorbidity and a potentially better prognosis or the prospect of transplantation remains unanswered. This may affect even a quarter of the entire population. Typically, such patients are treated with standard hemodialysis using high-flux membranes. The question arises whether it is worth using high-efficiency therapies and which of them is best tolerated by them. Apart from the obvious importance of survival outcome, the quality of life of patients and their tolerance of dialysis treatments should be taken into account [16,17,18]. Evidence-based medicine did not provide accurate recommendations about the best strategy to provide patients with a greater comfort of dialysis treatment. Therefore, therapy needs to be formulated and personalized, according to the heterogeneity of patients, based on their dominant co-morbidities, clinical characteristics and existing biochemical disorders. The individualization of treatment is based on the choice of dialysis techniques, dialysis membrane, the possibility of automatic regulation and profiling of ultrafiltration, sodium and potassium concentration and temperature in the dialysis bath, which is discussed in detail elsewhere [19].

Maintenance HD patients have a high burden of symptoms that negatively affect their quality of life [20]. Post-dialysis fatigue, intra-dialytic hypotension, cramps and dizziness are the most common symptoms reported by patients [21]. Post-dialysis fatigue and a lack of energy interfere with daily life and are also predictors of mortality [22]. Patients treated with standard HD report average DRT in the range of 2–4 h, with approximately 25% reporting DRT greater than 6 h [23,24]. In the FRENCHIE (French Convective versus Hemodialysis in Elderly) study, 25.9% of patients reported at least one AE during a dialysis session and 20.6% of patients had asymptomatic hypotension [25]. Moreover, patients may prioritize outcomes differently than those set by medical professionals. Focusing on the tolerance of the dialysis procedure and the comfort of life, we compared in the study various high-efficiency dialysis techniques used in the group of patients in whom these therapies are not commonly used. For an objective assessment of intradialytic stability, we used the method of impedance cardiography for real-time hemodynamic measurements.

Convective-based high-efficiency dialytic modalities, including online HDF, have been proposed as an alternative capable of relieving most intradialytic AEs and improving patient outcomes. HDF, used in various modes, including POST-HDF, PRE-HDF and MIX-HDF, provides a more effective removal of soluble middle molecular weight toxins and protein-bound compounds than conventional S-HD [1]. Other potential mechanisms underlying these effects are: (i) better biocompatibility due to the combined use of biocompatible membranes and ultrapure/sterile fluids, which results in a reduction in systemic inflammatory response; and (ii) a favorable impact of HDF on intradialytic hypotensive episodes due to a higher sodium mass transfer and mode-specific thermal effects [26]. Several previous studies investigating the influence of convection-based methods on intradialytic tolerance have yielded conflicting results. The FRENCHIE study compared high-flux HD and POST-HDF in terms of intradialytic tolerance in elderly chronic HD patients (over age 65) and reported significant differences between treatments with fewer episodes of intradialytic symptomatic hypotension and muscle cramps in POST-HDF [25]. Similar conclusions can be drawn from the results of the ESHOL trail [27]. However, in some studies, no improvement was observed in terms of intradialytic tolerance when switching therapy from S-HD to HDF [21,28,29] and some even indicate deterioration. For instance, in the crossover study by Smith J. et al., POST- HDF was associated with an increased rate of symptomatic hypotension compared to S-HD (8.0% vs. 5.3%) and intradialytic tendency to clotting (1.8% vs. 0.7%) [11]. The inclusion criteria we used are probably responsible for the fact that no episodes of intradialytic hypotension were recorded during any procedure in our study. We did not note any significant differences in dialysis tolerance between individual treatments, although some differences were clearly visible. POST-HD was the worst tolerated procedure. At least one AE was observed in almost 42% of POST-HDF sessions. The largest number of clotting events is noteworthy, which is fully understandable considering the highest degree of hemoconcentration during POST-HDF in the dialyzer, increasing the viscosity of the blood before fluid substitution, which results in the deposition of plasma proteins on the membrane surface, the clogging of membrane pores, an increased transmembrane pressure and an occlusion of dialyzer blood channels [30]. PRE-HDF resolves this problem but requires about three times more replacement fluid than POST-HDF. This reduces the risks of clotting and protein deposition and allows much higher ultrafiltration rates of up to 100% of the blood flow rate which can be far lower than in POST-HDF. The cooling effect of replacement solution in large volumes during PRE-HDF may help maintain hemodynamic stability as well [31]. During the PRE-HDF conducted in our study group, we observed fewer adverse symptoms than during POST-HDF. Locatelli et al. demonstrated 54% less intradialytic hypotension events in patients who were treated with PRE-HDF in comparison with a low-flux HD [32]. MIX-HDF is the least frequently used in clinical practice; hence, there is less tolerance studies on this method. In one of the few studies, symptomatic intradialytic hypotension episodes and other AEs occurred similarly in the MIX-HDF and PRE-HDF [33].

Small observational studies indicate that HDx may result in better treatment tolerance than standard HD with less dialysis hypotension and a reduction in DRT [34,35]. Other studies indicate that HDx use may be effective in reducing symptoms of restless leg syndrome, dialysis pruritus and improve quality of life [36,37]. It may be that removing a wider range of toxins, including large middle toxins, accounts for some of these benefits [38]. Compared to HDF, HDx does not increase transmembrane pressure, thus providing minimal stress to the filter [3]. Importantly, the HDX treatment is technically the simplest to perform among the high-efficiency methods, similar to standard hemodialysis, which may also affect the course of the procedure, with fewer complications and AEs. What is noteworthy in our study is that the number of observed and reported AEs was the lowest during HDx.

To the best of our knowledge, there are no studies comparing all high-efficiency dialysis modalities in the context of intradialytic tolerance, only individual small studies comparing PRE-HDF, only in relation to POST-HDF and HDx, and did not show any differences [39,40,41]. Our study seems to be pioneering in this respect, especially if we take into account the population in which the study was conducted. The lowest frequency of all AEs was observed with HDx (25%) and PRE-HDF (29%), although the differences did not prove to be statistically significant. There were no incidents of symptomatic intradialytic hypotension during any treatment modality. Our patients were characterized by strong cardiovascular stability. The use of impedance cardiography provided us with an indirect insight into cardiac output, blood viscosity and autonomic activity, as sympathetic stimulation constricts peripheral arteries and increases vascular resistance. In line with previous observations, CI decreased while SVRI increased during all methods used [42]. Of note, the CI AUC and SVRI AUC were not statistically different between all modalities, which indicates similar hemodynamic stability during the tested treatments.

Yet, another interesting patient outcome measure that we tracked in our study was the length of DRT. The length of DRT is a recent and reliable method of post-dialysis fatigue assessment, an important patient-reported complaint that affects their quality of life and restricts the ability to perform their daily activities [43]. Davenport et al. found that the DRT ≥1 h may be present in more than 75% of HD patients [44]. Most importantly, evidence from the DOPPS study has suggested an association between longer DRT and increased mortality [24]. Thus far, no convincing evidence has been obtained that dialysis methods based on convection, i.e., HDF, shortens the length of DRT [44,45]. There were also no differences in DRT and self-reported intradialytic symptoms with differing convection volumes during HDF [46]. Despite the lack of statistical significance, our results suggest that PRE-HDF may contribute to shortening post-dialysis fatigue more effectively than other compared therapies in the population that was the subject of our study. This improvement concerned both an increase in the percentage of patients who reported a return to well-being immediately after the dialysis, as well as a shortening of DRT in those for whom it required a longer time (Table 4). The analysis of the potential factors responsible for this phenomenon was beyond the scope of our study, but cooling effect of replacement solution in large volumes during PRE-HDF may be at least partially involved [31].

Our study has several strengths: (i) the choice of crossover design was made in order to abrogate the influence of interpatient variability; (ii) a detailed analysis of the variability of hemodynamic parameters over time was performed; (iii) the patients’ hydration status was measured and did not differ during individual treatments; (iv) basic dialysis parameters have been unified for all treatment modalities; (v) the high-volume nature of HDF, known to provide the best long-term prognosis, was assured during study. On the other hand, we are aware of the limitations of our study. We had only one woman in our study group, which may raise questions about its homogeneity, given the differences in body composition. However, the exclusion of female participants is a recognized problem in many nephrological studies and we decided against it [47]. The study included only relatively young patients with low comorbidities, who constitute the vast minority in dialysis centers. This means that the study results cannot be generalized to the entire dialysis population. On the other hand, such an approach allowed for the exclusion of most factors that might influence AEs except for the treatment modality (for example, diabetic neuropathy, atherosclerosis, heart failure or malnutrition). Another limitation is the single-center study design. The “center effect” is a well-known problem in studies about dialysis, secondarily to an endless list of aspects related to the clinical and nursing management of the dialysis session. We are convinced that the crossover design of the study should mitigate such a bias to a certain extent. The important limitation is also the small size of the study group. This is the cost that should be paid when eligibility criteria are set to control for many confounders. Taking all these limitations into account, one should be aware that only exploratory conclusions should be drawn.

In conclusion, the study did not find any significant differences in intradialytic AEs and DRT between standard high-flux HD and four high-efficacy HD modalities, including PRE-HDF, MIX-HDF, POST-HDF and HDx. However, the study results may suggest that tolerance of dialysis session and post-dialysis fatigue may vary in some patients when using different high-efficacy modalities. This indicates the necessity of individualizing HD therapy also in relatively young patients with low comorbidity.

## Figures and Tables

**Table 1:** Characteristics of the study group.

Gender (men/women)	11/1
Causes of ESRD (*n*/%)	
Autosomal dominant polycystic kidney disease	4/33.4
Glomerulonephritis (primary or secondary)	3/25.0
Hypertensive nephropathy	2/16.7
Renal malformation	1/8.3
Interstitial nephropathy Other	1/8.3 1/8.3
Age (years)	52.5 (15.5)
AACI (points)	4.5 (2.2)
Dialysis vintage (months)	42.5 (31.04)
Body mass index (kg/m^2^) Weight (kg) spKt/V_urea_ Hemoglobin (g/dL) Albumin (g/L)	23.8 (3.6) 73.7 (14.2) 1.5 (0.3) 10.9 (0.9) 33.1 (4.9)

ESRD: end-stage renal disease; AACI: Age-Adjusted Charlson Comorbidity Index.

**Table 2:** Delivered dialysis parameters, systemic blood pressure and hydration status parameters.

	S-HD	HDX	PRE-HDF	MIX-HDF	POST-HDF	*p*
Time min	240	240	240	240	240	NA
Blood flow mL/min	350	350	350	350	350	NA
Dialysate flow mL/min	500	500	500	500	500	NA
Ultrafiltration mL	2.12 (0.74)	2.33 (0.62)	2.45 (0.8)	2.29 (0.74)	2.19 (0.52)	*p* = 0.6
Ultrafiltration/dry weight %	0.028	0.032	0.034	0.031	0.029	*p* = 0.56
Total convection L	NA	NA	61.5 (7.2)	47.1 (11.4)	25.6 (3.8)	NA
SBP _predialysis_ mmHg	147.7 (27.5)	144.1 (20.3)	147.7 (26.6)	147.3 (20.3)	144.3 (22.4)	*p* = 0.95
DBP _predialysis_ mmHg	88.5 (18.8)	88.3 (16.9)	89.9 (20.4)	89.9 (16.4)	86.1 (18.0)	*p* = 0.93
TBW _predialysis_ l	39.76 (8.04)	41.64 (11.65)	39.05 (6.84)	40.15 (7.32)	39.7 (8.4)	*p* = 0.93
TBW _postdialysis_ l	38.17 (8.03)	40.46 (12.51)	37.5 (6.97)	38.56 (7.29)	37.44 (8.24)	*p* = 0.85
ECW _predialysis_ l	19.1 (3.2)	19.9 (3.3)	20.1 (3.5)	19.3 (3.5)	18.9 (3.2)	*p* = 0.74
ECW _postdialysis_ l	17.2 (3.1)	17.43 (3.1)	17.38 (2.9)	18.2 (5.7)	16.7 (2.9)	*p* = 0.77
ICW _predialysis_ l	21.31 (5.6)	23.3 (7.5)	22.2 (5.1)	20.7 (4.2)	20.8 (5.4)	*p* = 0.62
ICW _postdialysis_ l	21.33 (5.7)	24.5 (8.8)	24.2 (6.5)	21.2 (4.7)	20.7 (5.5)	*p* = 0.17

Note: Ultrafiltration: the fluid removal during the session; total convection: the total volume of convection during the session, which is the sum of the patient’s dehydration volume and the volume of the replacement fluid administered; SBP: systolic blood pressure; DBP: diastolic blood pressure; TBW: total body water; ECW: extracellular water; ICW: intracellular water.

**Table 3:** Systemic blood pressure and area under the curve (AUC) of hemodynamic parameters.

	S-HD	HDX	PRE-HDF	MIX-HDF	POST-HDF	*p*
SBP 1st _min_ mmHg	145.8 (24.6)	139.1 (17.2)	143.4 (22.6)	141.2 (18.0)	137.9 (21.9)	*p* = 0.75
SBP _240 min_ mmHg	142.5 (35.5)	142.2 (28.3)	138.7 (35.7)	140.6 (35.5)	138.8 (29.2)	*p* = 0.98
DBP 1st _min_ mmHg	87.0 (17.5)	86.3 (14.3)	86.1 (16.7)	87.9 (16.6)	83.4 (15.8)	*p* = 0.85
DBP _240 min_ mmHg	85.7 (17.1)	89.1 (21.3)	84.9 (17.3)	85.3 (20.5)	87.3 (18.7)	*p* = 0.91
AUC SBP	323 816.6 (72,781.6)	318 930.3 (61,252.4)	316 602.0 (68,292.8)	305 190.3 (76,556.9)	313 049.4 (80,028.1)	*p* = 0.8
AUC DPB	194,716.4 (37,664.1)	192,651.0 (53,530.6)	194,253.7 (33,794.1)	190,661.9 (44,971.5)	191,900.7 (44,991.7)	*p* = 0.88
AUC MAP	237,748.1 (46,888.6)	230,184.4 (59,405.4)	235,096.4 (42,932.1)	231,486.3 (53 996.6)	234,209.5 (47,871.5)	*p* = 0.23
AUC CI	6559.2 (1439.5)	6770.9 (1271.3)	6512.5 (1256.4)	6093.9 (1282.4)	6680.9 (1652.9)	*p* = 0.65
AUC SVRI	6,176,119.3 (1,325,662.8)	6,456,193.4 (1,473,702.1)	6,256,567.9 (999,108.8)	7,075,464.9 (1,930,210.7)	6,301,942.5 (1,337,688.1)	*p* = 0.34

SBP: systolic blood pressure; DBP: diastolic blood pressure; MAP: mean arterial pressure; CI: cardiac index; SVRI: systemic vascular resistance index.

**Table 4:** Adverse events (% events per sessions) and dialysis recovery time (DRT).

	S-HD	HDX	PRE-HDF	MIX-HDF	POST-HDF	*p*
Symptomatic hypotension n	0	0	0	0	0	*p* = 1.0
AEs potentially related to BP/fluid shifts n	0	1	1	2	4	*p* = 0.39
AEs potentially not related to BP/fluid shifts n	7	4	5	5	2	*p* = 0.47
Intradialytic clotting events n	1	1	2	2	4	*p* = 0.51
All AEs *n* (%)	8 (33.3%)	6 (25%)	7 (29.2%)	9 (37.5%)	10 (41.7%)	*p* = 0.76
Immediate DRT *n* (%)	11 (45.8%)	12 (50%)	15 (62.5%)	9 (37.5%)	10 (41.7%)	*p* = 0.10
Delayed DRT min	360.0 (180–720)	180 (120–390)	60 (30–600)	360 (180–360)	390 (60–720)	*p* = 0.37

Note: AEs: adverse events; values are given as number of events (percentage). Multiples of the same episodes within 1 session were treated as a single event. All AEs: all AEs reported by patients and reported in the table, including clotting events; DRT: dialysis recovery time.

## Data Availability

The data are available from the corresponding authors upon reasonable request.
